# Minimalist module analysis for fault detection and localization

**DOI:** 10.1038/s41598-021-02676-3

**Published:** 2021-12-07

**Authors:** Zhijiang Lou, Youqing Wang, Shan Lu, Pei Sun

**Affiliations:** 1 Institute of Intelligence Science and Engineering, Shenzhen Polytechnic, Shenzhen, 518055 China; 2 College of Electrical Engineering and Automation, Shandong University of Science and Technology, Qingdao, 266590 China

**Keywords:** Chemical engineering, Applied mathematics, Statistics

## Abstract

Traditional multivariate statistical-based process monitoring (MSPM) methods are effective data-driven approaches for monitoring large-scale industrial processes, but have a shortcoming in handling the redundant correlations between process variables. To address this shortcoming, this study proposes a new MSPM method called minimalist module analysis (MMA). MMA divides process data into several different minimalist modules and one more independent module. All variables in the minimalist module are strongly correlated, and no redundant variables exist; therefore, the extracted feature components in one minimalist module will not be disturbed by noise from the other modules. This study also proposes new monitoring indices and a fault localization strategy for MMA, and simulation tests demonstrate that MMA achieves superior performance in fault detection and localization.

## Introduction

Multivariate statistical-based process monitoring (MSPM) methods^1–4^, e.g., principal component analysis (PCA)^5, 6^, partial least squares (PLS)^7, 8^, and canonical correlation analysis (CCA)^9, 10^, are effective data-driven approaches for monitoring large-scale industrial processes. The main idea of MSPM is analyzing the correlation between process variables and extracting the feature components for the construction of statistical indices.

MSPM has been a research hotspot for many years, and a large number of relevant studies are published each year. In recent years, studies have focused on improving the existing methods to deal with process characteristics such as nonlinear, non-Gaussian, and dynamic features. For example, Ge et al.^11^ combines the multivariate linear Gaussian state-space model with MSPM for handling the dynamic feature during a process; Du et al.^12^ proposed the Gaussian distribution transformation (GDT)-based monitoring method for handling the non-Gaussian feature; and Lou et al.^13^ combined artificial neural networks with PCA, and proposed a new neural component analysis for handling nonlinear features. Meanwhile, Zhou et al.^14^ proposed a nonlinear key performance indicator (KPI) strategy for the PLS algorithm.

Because MSPM can compress the high-dimensional data into two or three statistical indices, it is a convenient tool for detecting the abnormal condition in the whole process object. To address the fault localization problem, the contribution plot method^15, 16^ was proposed for MSPM, which calculates the contribution of each variable of the original data set and picks the variables with high contributions as fault sources. Most studies on MSPM use the contribution plot as a basic algorithm tool^17, 18^, and a few studies have proposed improved versions of the MSPM method that cannot use the traditional contribution plot directly (examples include the kernel PCA^19^ and robust PCA^20^).

However, according to actual simulation test results, MSPM is insensitive to specific faults, and the contribution plot method may mistakenly diagnose normal variables as a fault source. The reason for this phenomenon is that the traditional MSPM methods are based on the correlations between all process variables, and some correlations can be deduced by others, which means that these correlations are redundant. As such, the feature components extracted by traditional MSPM methods contain information from many process variables, and hence, are also disturbed by noises from these variables; therefore, traditional MSPM methods are insensitive to specific faults. In addition, the redundant correlations may mislead the contribution plot method, which results in incorrect localization of faults.

**Figure 1:**
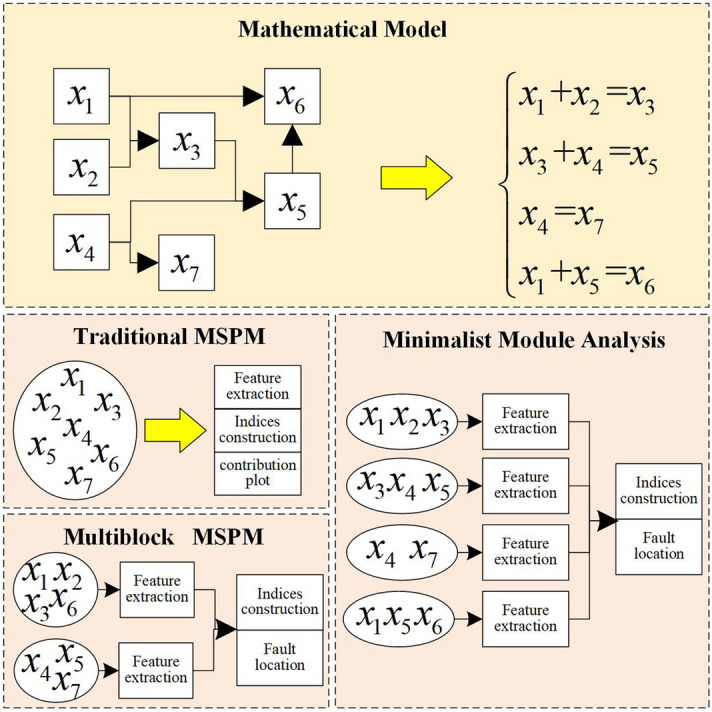
The traditional multivariate statistical-based process monitoring (MSPM) methods, multiblock MSPM, and minimalist module analysis.

For handling these problems, multiblock MSPM methods, such as consensus PCA (CPCA)^21^, multiblock PLS (MBPLS)^18^, and hierarchical PLS (HPLS)^22^, are proposed for reducing the number of variables and improving the interpretability of multivariate models. The main idea of multiblock MSPM methods is dividing the process variables into several blocks and combining the monitoring result of each block. However, block division is still an open problem in academic and engineering fields. Though Slama had given a general guideline “blocks should correspond as closely as possible to distinct units of the process where all the variables within a block or process unit may be highly coupled, but where there is minimal coupling among variables in different blocks”^18^, this rule is inappropriate for large-scale industrial processes, because (a) in large-scale industrial processes, variables in different process units are still highly coupled; (b) variables in the same unit may be unrelated. In addition, for multiblock MSPM methods, one variable only belongs to one block, as such, the rest blocks may lose key input variables, which causes large model error. For example, for model in Fig. 1, it’s hard to divide the process variables into two or more blocks: when $x_{3}$ is allocated to block 2, then blocks 1 loses information of $x_{3}$. Besides, it’s difficult to divide the blocks with traditional data-driven method, and hence many multiblock MSPM methods demand the process prior knowledge for block division^23^.

To eliminate the influence of the redundant correlations among process data, this paper proposes a novel MSPM method called minimalist module analysis (MMA). All variables in the minimalist module are strongly correlated, and no redundant variables exist. As shown in Fig. 1, MMA just analyzes the correlations between variables in the same module, and hence the extracted feature components are not disturbed by the noise from the other modules. In addition, the modularization analysis results can provide more useful information for fault localization.

The difference between MMA and the multiblock MSPM methods are as follows: first, for MMA, each variable may belong to more than one modules ($x_{1}$ belongs to two modules in Fig. 1), so each module represents one complete correlation without information loss; second, for MMA, module division is based on statistics analysis rather than the process prior knowledge, which is consistent with the data-driven feature of MSPM; third, each module only contain one correlation in MMA, and each block in the multiblock MSPM methods may contain more than one correlations.

The main innovations of this study are as follows. First, we propose a modularization method based on singular value decomposition (SVD)^24^ and particle swarm optimization (PSO)^25^, which can divide the process variables into different minimalist modules and an independent module. Then, we propose new monitoring indices for each module. In addition, we propose a new fault localization strategy for MMA.

According to a survey paper^1^, PCA is the most commonly used MSPM method. As such, this paper focuses on the comparison of MMA and PCA; our conclusion is also applicable to other algorithms, such as PLS and CCA. The simulation tests in a mathematical model and the Tennessee Eastman (TE) process^26^ show that MMA can successfully obtain the minimalist modules; moreover, it achieves much better performance than the traditional MSPM methods in fault detection and fault localization.

The remainder of this paper is organized as follows. In “Methods” section, we briefly review some concepts of classical PCA and the contribution plot method, and assess the defects of these methods. “Minimalist module analysis (MMA)” section then proposes MMA for process monitoring, and introduces some details. “Simulation study of MMA” section analyzes the characteristics of MMA, and compares this method with PCA by conducting tests on a mathematical model. “Fault detection in the Tennessee Eastman process” section compares MMA with other improved MSPM methods in the TE process. Lastly, “Conclusions” section summarizes the contributions of this paper, and discusses some directions for future studies.

## Methods

### Principal component analysis (PCA)

PCA decomposes the data matrix ${\mathbf{X}} \in {\mathbf{R}}^{n \times s}$ (where *n* is the number of samples, and *s* is the number of variables) into a transformed *k* subspace of reduced dimensions as follows:

1 $${\mathbf{X}} = {\mathbf{TP}}^{{\mathbf{T}}} + {\mathbf{E}} = \hat{\mathbf{X}} + {\mathbf{E}},$$

where ${\mathbf{T}} \in {\mathbf{R}}^{n \times k}$ refers to the score matrix, which is an orthogonal matrix; ${\mathbf{P}} \in {\mathbf{R}}^{s \times k}$ refers to the loading matrix, and it is orthonormal; and ${\mathbf{E}} \in {\mathbf{R}}^{n \times s}$ is the residual matrix. To obtain the loading matrix ${\mathbf{P}}$, one should firstly calculate the covariance matrix:

2 $${{\varvec{\Xi}}} = \frac{1}{n - 1}{\mathbf{X}}^{T} {\mathbf{X}}$$

Then, ${{\varvec{\Xi}}}$ can be presented by singular value decomposition (SVD) as follows:

3 $${{\varvec{\Xi}}} = {\mathbf{P}}_{0}^{T} {\mathbf{\Lambda P}}_{0} ,$$

where $${\mathbf{\Lambda = }}\left[ {\begin{array}{*{20}l} {\lambda_{1} } \hfill & 0 \hfill & 0 \hfill & 0 \hfill \\ 0 \hfill & {\lambda_{2} } \hfill & 0 \hfill & 0 \hfill \\ 0 \hfill & 0 \hfill & \ddots \hfill & 0 \hfill \\ 0 \hfill & 0 \hfill & 0 \hfill & {\lambda_{s} } \hfill \\ \end{array} } \right]$$ ($\lambda_{1} \ge \lambda_{2} \ge \cdots \lambda_{s} \ge 0$) is a diagonal matrix. Matrix ${\mathbf{P}}$ is actually columns of **P**_0_ associated with the *k* largest eigenvalues, and *k* is determined by cumulative percent variance (CPV)^27^ as follows:

4 $$CPV = \sum\limits_{i = 1}^{k} {\lambda_{i} } /\sum\limits_{i = 1}^{s} {\lambda_{i} \times 100\% \ge \varepsilon ,}$$

where $\varepsilon$ is a parameter usually set to 85%. When *CPV* is larger than $\varepsilon$, we take *k* as the number of the principal components (PCs).

Then, two statistics are constructed to monitor the new process data sample ${\mathbf{x}} \in {\mathbf{R}}^{1 \times s}$ as follows:

5 $$\left\{ \begin{gathered} T^{2} = {\mathbf{xP}}({{\varvec{\Lambda}}}_{k} )^{ - 1} {\mathbf{P}}^{T} {\mathbf{x}}^{T} \hfill \\ SPE = ({\mathbf{x}} - {\hat{\mathbf{x}}})({\mathbf{x}} - {\hat{\mathbf{x}}})^{T} \hfill \\ \end{gathered} \right.,$$

where ${\hat{\mathbf{x}}} = {\mathbf{TP}}^{T} = {\mathbf{xPP}}^{T}$ and $${{\varvec{\Lambda}}}_{k} { = }\left[ {\begin{array}{*{20}c} {\lambda_{1} } & 0 & 0 & 0 \\ 0 & {\lambda_{2} } & 0 & 0 \\ 0 & 0 & \ddots & 0 \\ 0 & 0 & 0 & {\lambda_{k} } \\ \end{array} } \right]$$ $\left( {\lambda_{1} \ge \lambda_{2} \ge \cdots \lambda_{k} \ge 0} \right)$. The thresholds for the two indices, $\delta_{{T^{2} }}$ and $\delta_{SPE}$, can be found in reference^28^.

### Contribution plot

The contributions to *SPE* are calculated as follows:

6 $$Con SPE_{j} = ({\mathbf{x}}_{j} - {\hat{\mathbf{x}}}_{j} )({\mathbf{x}}_{j} - {\hat{\mathbf{x}}}_{j} )^{T} ,$$

where ${\mathbf{x}}_{j}$ and ${\hat{\mathbf{x}}}_{j}$ are the *j*th columns of **x** and ${\hat{\mathbf{x}}}$, respectively. The contributions to $T^{2}$ are calculated as follows:

7 $$ConT_{j}^{2} = \sum\limits_{i = 1}^{k} {\left( {{\mathbf{x}}_{j} - {\hat{\mathbf{x}}}_{j} } \right){\mathbf{P}}_{j,i} \lambda_{i}^{ - 1} } {\mathbf{P}}_{i}^{T} {\mathbf{x}}^{T} ,$$

where **P**_*i*_ is the *i*th column of **P**, and **P**_*j,i*_ is the element in the *j*th column and *i*th row.

The role of the contribution plots to fault isolation is to indicate which of the variables are related to the fault rather than to reveal the actual cause of it. In general, variables with a higher contribution have a closer relationship with the fault source. The thresholds of and can be obtained by kernel density estimation^29^.

### Drawback of PCA and contribution plot method

#### **Theorem**

*The redundant variables introduce extra noise into the principal components (PCs)*.

#### ***Proof***

Assume ${\mathbf{X}}_{1} \in {\mathbf{R}}^{n \times s}$ are the variables belonging to a minimalist module, which can be full-rank decomposed as

8 $${\mathbf{X}}_{1} = {\mathbf{T}}_{0} {\mathbf{P}}_{0}^{T} ,$$

where ${\mathbf{T}}_{0} \in {\mathbf{R}}^{n \times s}$ and ${\mathbf{P}}_{0} \in {\mathbf{R}}^{s \times s}$ Matrix ${\mathbf{X}}_{2} \in {\mathbf{R}}^{{n \times s^{\prime}}}$ are the redundant variables that can be presented as the linear combination of **X**_1_ as follows:

9 $${\mathbf{X}}_{2} = {\mathbf{X}}_{1} {\mathbf{R}} + {\mathbf{W}},$$

where ${\mathbf{R}} \in {\mathbf{R}}^{{s \times s{\prime }}}$. is the linear transformation matrix, and ${\mathbf{W}} \in {\mathbf{R}}^{{n \times s{\prime }}}$ is noise belonging to **X**_2_. In this paper, we assume that each measurement variable contains independent sensor noise, and hence, *rank*(**W**) = *s*′.

Taking $${\mathbf{X}} = \left[ {\begin{array}{*{20}c} {{\mathbf{X}}_{1} } & {{\mathbf{X}}_{2} } \\ \end{array} } \right]$$, one obtains

10 $${\mathbf{X}} = {\mathbf{T}}_{0} {\mathbf{P}}_{0}^{T} \left[ {\begin{array}{*{20}c} {\mathbf{I}} & {\mathbf{R}} \\ \end{array} } \right] + \left[ {\begin{array}{*{20}c} {\mathbf{0}} & {\mathbf{W}} \\ \end{array} } \right].$$

Part $${\mathbf{T}}_{0} {\mathbf{P}}_{0}^{T} \left[ {\begin{array}{*{20}l} {\mathbf{I}} \hfill & {\mathbf{R}} \hfill \\ \end{array} } \right]$$ can be full-rank singular value decomposed as

11 $${\mathbf{T}}_{0} {\mathbf{P}}_{0}^{T} \left[ {\begin{array}{*{20}c} {\mathbf{I}} & {\mathbf{R}} \\ \end{array} } \right] = {\mathbf{T}}_{1} {\mathbf{P}}_{1}^{T} ,$$

where ${\mathbf{T}}_{1} \in {\mathbf{R}}^{{n \times \left( {s + s^{\prime}} \right)}}$, *rank*(**T**_1_) = *rank*(**T**_0_), and ${\mathbf{P}}_{1} \in {\mathbf{R}}^{{(s + s{\prime }) \times \left( {s + s^{\prime}} \right)}}$. Hence, one obtains

12 $${\mathbf{X}} = {\mathbf{T}}_{1} {\mathbf{P}}_{1}^{T} + \left[ {\begin{array}{*{20}c} {\mathbf{0}} & {\mathbf{W}} \\ \end{array} } \right]{ = }\left( {{\mathbf{T}}_{1} { + }\left[ {\begin{array}{*{20}c} {\mathbf{0}} & {\mathbf{W}} \\ \end{array} } \right]{\mathbf{P}}_{1} } \right){\mathbf{P}}_{1}^{T} .$$

Taking $${\mathbf{P}}_{1} = \left[ {\begin{array}{*{20}c} {{\mathbf{P^{\prime}}}_{1} \in {\mathbf{R}}^{{s \times \left( {s + s^{\prime}} \right)}} } \\ {{\mathbf{P^{\prime\prime}}}_{1} \in {\mathbf{R}}^{{s^{\prime} \times \left( {s + s^{\prime}} \right)}} } \\ \end{array} } \right]$$,

13 $${\mathbf{X}} = \left( {{\mathbf{T}}_{1} { + }{\mathbf{WP^{\prime\prime}}}_{1} } \right){\mathbf{P}}_{1}^{T} .$$

Because part $\left( {{\mathbf{T}}_{1} { + }{\mathbf{WP}}_{1}^{{\prime \prime }} } \right)$ is non-orthogonal in most situations, we introduce another orthonormal matrix ${\mathbf{Q}} \in {\mathbf{R}}^{{\left( {s + s^{\prime}} \right) \times \left( {s + s^{\prime}} \right)}}$, which makes

14 $$\left\{ {\begin{array}{*{20}l} {{\mathbf{X}} = {\mathbf{T}}_{2} {\mathbf{P}}_{2}^{T} } \hfill \\ {{\mathbf{T}}_{2} {\text{ = }}\left( {{\mathbf{T}}_{1} {\text{ + }}{\mathbf{WP}}_{1}^{{\prime \prime }} } \right){\mathbf{Q}}} \hfill \\ {{\mathbf{P}}_{2} {\text{ = }}{\mathbf{P}}_{1} {\mathbf{Q}}} \hfill \\ \end{array} } \right.$$

It should be noted that when $\left( {{\mathbf{T}}_{1} { + }{\mathbf{WP}}_{1}^{{\prime \prime }} } \right)$ is orthogonal, then **Q** = **I**.

PCA picks the *k* largest components of **T**_2_ as PCs, and we denote them as ${\mathbf{T}}_{k} \in {\mathbf{R}}^{n \times k}$. Then,

15 $${\mathbf{T}}_{k} { = }\left( {{\mathbf{T}}_{1} { + }{\mathbf{WP}}_{1}^{{\prime \prime }} } \right){\mathbf{Q}}_{k} = {\mathbf{T}}_{1} {\mathbf{Q}}_{k} + {\mathbf{WP}}_{1}^{{\prime \prime }} {\mathbf{Q}}_{k} ,$$

where ${\mathbf{Q}}_{k} \in {\mathbf{R}}^{{(s + s^{\prime}) \times k}}$ is the corresponding *k* columns of ${\mathbf{Q}}$. Taking ${{\varvec{\Pi}}}{ = }{\mathbf{P}}_{1}^{{\prime \prime }} {\mathbf{Q}}_{k} \in {\mathbf{R}}^{{s^{\prime} \times k}}$, and because ${\mathbf{P^{\prime\prime}}}$ and ${\mathbf{Q}}_{k}$ are parts of orthonormal matrices ${\mathbf{P}}_{1}$ and ${\mathbf{Q}}$, one obtains ${{\varvec{\Pi}}} \ne {\mathbf{0}}$($rank\left( {{\varvec{\Pi}}} \right) \ne 0$) unless the exceptionally rare situation that all columns of **Q**_*k*_ belong to the column set of ${\mathbf{P^{\prime}}}_{1}^{T}$. As $rank\left( {\mathbf{W}} \right){ + }rank\left( {{\varvec{\Pi}}} \right) > s^{\prime}$, one obtains ${\mathbf{WP}}_{1}^{{\prime \prime }} {\mathbf{Q}}_{k} \ne 0$.

As such, **T**_*k*_ is influenced by **W**, and the redundant variables **X**_2_ introduce extra noise **W** into the principal components (PCs). This finishes the proof. Based on the **Theorem**, one finds that PCA is not good at handling process data with redundant variables.

**Figure 2:**
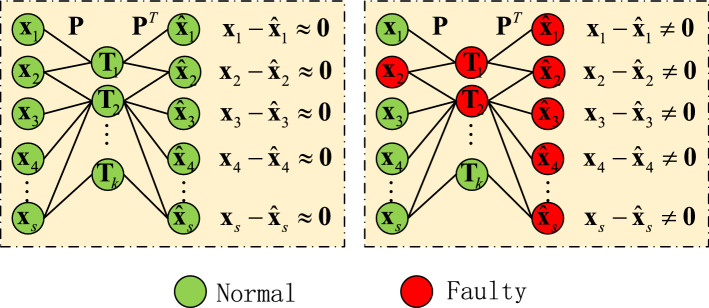
Fault propagation from original data to reconstructed data.

As for the contribution plot method, according to Eqs. (6) and (7), it is based on the difference between **x** and ${\hat{\mathbf{x}}}$. As shown in Fig. 2, when a fault occurs in a specific variable ${\mathbf{x}}_{j}$, (a) according to equation ${\mathbf{T}}{ = }{\mathbf{xP}}$, the relevant principal components are faulty; (b) according to equation ${\hat{\mathbf{x}}}{ = }{\mathbf{TP}}^{T}$, most reconstructed variables are faulty. As such, in a practical engineering application, it is hard to locate the source fault by the contribution plot method because too many variables’ contribution indices alarm the fault.

### Section summary

In sum, to eliminate the noise disturbance in the redundant variables, and to improve the fault localization ability, we develop a new monitoring algorithm based on the minimalist module and propose a corresponding fault localization strategy in “Minimalist module analysis (MMA)” section.

## Minimalist module analysis (MMA)

**Figure 3:**
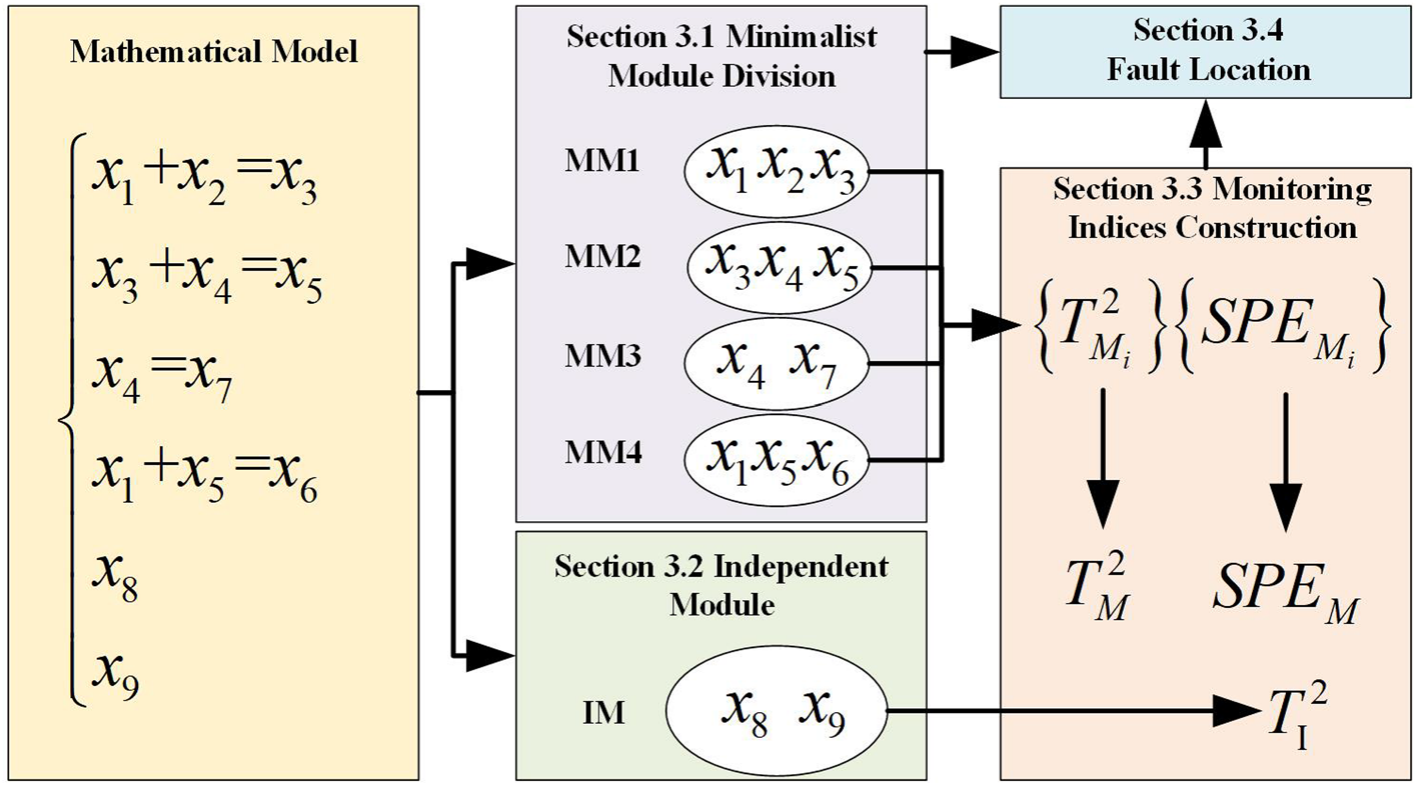
Content of this “Minimalist module analysis (MMA)” section.

The content of this section is listed in Fig. 3 below.

### Minimalist module division

Traditional PCA approaches focus on the *k* largest eigenvalues in matrix ${{\varvec{\Lambda}}}$, and the important information contained in the residual part is not used. When $\varepsilon$ is very small (e.g., 0.05), one obtains $\lambda_{j} \approx 0{\kern 1pt} \left( {j = k + 1.k + 2, \ldots ,s} \right)$. Taking **P**_*r*_ as the columns of **P**_0_ associated with the *s*-*k* smallest eigenvalues, one obtains

16 $${\mathbf{XP}}_{r} \approx {\mathbf{0}}.$$

We assume $${\mathbf{X}} = \left[ {\begin{array}{*{20}c} {x_{1} } & {x_{2} } & {x_{3} } \\ \end{array} } \right]$$, and $${\mathbf{P}}_{r} { = }\left[ {\begin{array}{*{20}c} {{\mathbf{P}}_{1,1} } & {{\mathbf{P}}_{1,2} } \\ {{\mathbf{P}}_{2,1} } & {{\mathbf{P}}_{2,2} } \\ {{\mathbf{P}}_{3,1} } & {{\mathbf{P}}_{3,2} } \\ \end{array} } \right]$$. Then,

17 $$\left\{ \begin{gathered} x_{1} {\mathbf{P}}_{1,1} { + }x_{2} {\mathbf{P}}_{2,1} { + }x_{3} {\mathbf{P}}_{3,1} \approx 0 \hfill \\ x_{1} {\mathbf{P}}_{1,2} { + }x_{2} {\mathbf{P}}_{2,2} { + }x_{3} {\mathbf{P}}_{3,2} \approx 0 \hfill \\ \end{gathered} \right..$$

Through the transformation of Eq. (17), one obtains

18 $$\begin{aligned} & \left( {x_{1} {\mathbf{P}}_{1,1} { + }x_{2} {\mathbf{P}}_{2,1} { + }x_{3} {\mathbf{P}}_{3,1} } \right){\mathbf{P}}_{1,2} { - }\left( {x_{1} {\mathbf{P}}_{1,2} { + }x_{2} {\mathbf{P}}_{2,2} { + }x_{3} {\mathbf{P}}_{3,2} } \right){\mathbf{P}}_{1,1} \\ & { = }x_{2} \left( {{\mathbf{P}}_{2,1} {\mathbf{P}}_{1,2} { - }{\mathbf{P}}_{2,2} {\mathbf{P}}_{1,1} } \right){ + }x_{3} \left( {{\mathbf{P}}_{3,1} {\mathbf{P}}_{1,2} { - }{\mathbf{P}}_{3,2} {\mathbf{P}}_{1,1} } \right) \approx 0. \\ \end{aligned}$$

As such, one then obtains

19 $$\left\{ \begin{gathered} {\tilde{\mathbf{P}}}_{r} = \left[ {\begin{array}{*{20}c} 0 \\ {{\mathbf{P}}_{2,1} {\mathbf{P}}_{1,2} { - }{\mathbf{P}}_{2,2} {\mathbf{P}}_{1,1} } \\ {{\mathbf{P}}_{3,1} {\mathbf{P}}_{1,2} { - }{\mathbf{P}}_{3,2} {\mathbf{P}}_{1,1} } \\ \end{array} } \right] = {\mathbf{P}}_{r} \left[ {\begin{array}{*{20}c} {{\mathbf{P}}_{1,2} } \\ {{ - }{\mathbf{P}}_{1,1} } \\ \end{array} } \right] \hfill \\ {\mathbf{X}}\tilde{\mathbf{P}}_{r} \approx {\mathbf{0}} \hfill \\ \end{gathered} \right.$$

Unlike ${\mathbf{P}}_{r}$, some elements of ${\tilde{\mathbf{P}}}_{r}$ are 0, and hence Eq. (19) can describe the relationship between *x*_2_ and *x*_3_ without considering *x*_1_. In Eq. (19), variable set $$\left[ {\begin{array}{*{20}l} {x_{2} } \hfill & {x_{3} } \hfill \\ \end{array} } \right]$$ is a minimalist module.

The flow of minimalist module division is as follows:

- Find a transformation matrix ${{\varvec{\Gamma}}} \in {\mathbf{R}}^{{\left( {s{ - }k} \right) \times \left( {s - k} \right)}}$ that maximizes the number of 0 elements in ${\tilde{\mathbf{P}}}_{r} = {\mathbf{P}}_{r} {{\varvec{\Gamma}}}$. This paper addresses this optimization problem by using the particle swarm optimization (PSO)^30^ algorithm as described below.

*Step 1* Set *num* = 1.

*Step 2* Take the $num^{th}$ column of **P**_*r*_ as ${{\varvec{\Psi}}}_{{\mathbf{1}}}$ and the remaining *s* − *k* − 1 columns as ${{\varvec{\Psi}}}_{{\mathbf{2}}}$. Solve the following optimization function by PSO:

20 $$\mathop {Minimize}\limits_{{{{\varvec{\Gamma}}}_{num} }} \left( {\left\| {{{\varvec{\Psi}}}_{1} - {{\varvec{\Psi}}}_{2} {{\varvec{\Gamma}}}_{num} } \right\|_{2} { - }\left\| {{{\varvec{\Psi}}}_{1} - {{\varvec{\Psi}}}_{2} {{\varvec{\Gamma}}}_{num} } \right\|_{\beta } } \right),$$

where $\left\| {{{\varvec{\Psi}}}_{1} - {{\varvec{\Psi}}}_{2} {{\varvec{\Gamma}}}_{num} } \right\|_{\beta }$ denotes the number of elements in interval $\left[ { - \beta ,\beta } \right]$ ($\beta$ is close to 0, such as 0.01).

*Step 3* If $num = s - k$, go to step 4; else, *num* = *num* + 1 and go to step 2.

*Step 4* $${\mathbf{\Gamma = {\rm I} - }}\left[ {\begin{array}{*{20}l} {{{\varvec{\Gamma}}}_{1} } \hfill & {{{\varvec{\Gamma}}}_{2} } \hfill & \ldots \hfill & {{{\varvec{\Gamma}}}_{s - k} } \hfill \\ \end{array} } \right]$$.

(b) Calculate ${\tilde{\mathbf{P}}}_{r} { = }{\mathbf{P}}_{r} {{\varvec{\Gamma}}}$, adjust each column of ${\tilde{\mathbf{P}}}_{r}$ to unit variance, and set all elements in interval $\left[ {{ - }\beta \beta } \right]$ to 0.
(c) Take the variables corresponding to non-zero element parameters in the *i*th ($i = 1,2, \ldots ,s - k$) column of ${\tilde{\mathbf{P}}}_{r}$ as the *i*th minimalist module (MMi).

#### ***Remark***

The form of the minimalist module is not unique, e.g. through the transformation of Eq. (17), one also obtains.

$$\begin{aligned} & \left( {x_{1} {\mathbf{P}}_{1,1} { + }x_{2} {\mathbf{P}}_{2,1} { + }x_{3} {\mathbf{P}}_{3,1} } \right){\mathbf{P}}_{3,2} { - }\left( {x_{1} {\mathbf{P}}_{1,2} { + }x_{2} {\mathbf{P}}_{2,2} { + }x_{3} {\mathbf{P}}_{3,2} } \right){\mathbf{P}}_{3,1} \\ & { = }x_{1} \left( {{\mathbf{P}}_{1,1} {\mathbf{P}}_{3,2} { - }{\mathbf{P}}_{1,2} {\mathbf{P}}_{3,1} } \right){ + }x_{2} \left( {{\mathbf{P}}_{2,1} {\mathbf{P}}_{3,2} { - }{\mathbf{P}}_{2,2} {\mathbf{P}}_{3,1} } \right) \approx 0, \\ \end{aligned}$$

and hence variable set $$\left[ {\begin{array}{*{20}c} {x_{1} } & {x_{2} } \\ \end{array} } \right]$$ is also a minimalist module. As such, the result of PSO may be different each time.

### Independent module

Each variable in the minimalist module is strongly correlated with other variables. As such, some variables, such as *x*_8_ and *x*_9_ in Fig. 3, are not included in the minimalist module group. Thus, these variables belong to the independent module.

### Monitoring indices construction

Each minimalist module can be monitored by the PCA algorithm independently. We assume that ${\tilde{\mathbf{X}}}_{i} \in {\mathbf{R}}^{{n \times \tilde{s}}}$ are data belonging to MMi. Then, $rank\left( {{\tilde{\mathbf{X}}}_{i} } \right) \in \tilde{s} - 1$ because each minimalist module represents one independent correlation, and hence the number of PCs for each minimalist module is fixed as $\tilde{s} - 1$. The monitoring indices of each module are calculated as

21 $$T_{{M_{i} }}^{2} { = }{{T_{i}^{2} } \mathord{\left/ {\vphantom {{T_{i}^{2} } {\delta_{{T_{i}^{2} }} }}} \right. \kern-\nulldelimiterspace} {\delta_{{T_{i}^{2} }} }},$$

and

22 $$SPE_{{M_{i} }} { = }{{\left( {{{SPE_{i} } \mathord{\left/ {\vphantom {{SPE_{i} } {T_{{M_{i} }}^{2} }}} \right. \kern-\nulldelimiterspace} {T_{{M_{i} }}^{2} }}} \right)} \mathord{\left/ {\vphantom {{\left( {{{SPE_{i} } \mathord{\left/ {\vphantom {{SPE_{i} } {T_{{M_{i} }}^{2} }}} \right. \kern-\nulldelimiterspace} {T_{{M_{i} }}^{2} }}} \right)} {\delta_{{SPE_{i} }} }}} \right. \kern-\nulldelimiterspace} {\delta_{{SPE_{i} }} }},$$

where $T_{i}^{2}$ and $SPE_{i}$, and $\delta_{{T_{i}^{2} }}$ and $\delta_{{SPE_{i} }}$ are the $T^{2}$ and *SPE* indices and the corresponding thresholds for MMi, respectively. Different from the traditional *SPE* index, *SPE*_*i*_ divides $T_{{M_{i} }}^{2}$ to eliminate the impact of $T_{{M_{i} }}^{2}$ on *SPE*_*i*_.

The indices for the whole process are

23 $$T_{M}^{2} { = }\sum\limits_{i = 1}^{s - k} {\left( {1 + \gamma * sign\left( {T_{{M_{i} }}^{2} - 1} \right)} \right)T_{{M_{i} }}^{2} } ,$$

and

24 $$SPE_{M} { = }\sum\limits_{i = 1}^{s - k} {\left( {1 + \gamma * sign\left( {SPE_{{M_{i} }} - 1} \right)} \right)SPE_{{M_{i} }} } ,$$

where $\gamma$ is a positive value (e.g., $\sqrt {s - k}$). As such, when some minimalist module detects the fault, then these two indices are much larger than their normal values. The threshold for both indices is $s - k$.

As for the variables in the independent module, they can be monitored by the $T^{2}$ index, which is denoted as $T_{I}^{2}$.

### Fault localization

For MMA, the fault localization rules are different for $T_{M}^{2}$, $SPE_{M}$, and $T_{I}^{2}$ indices.

- For the $T_{M}^{2}$ index, when $T_{{M_{i} }}^{2}$ is normal, then all related variables are normal. For example, in the mathematical model in Fig. 3, when $T_{{M_{1} }}^{2}$ and $T_{{M_{2} }}^{2}$ are faulty, and $T_{{M_{3} }}^{2}$ and $T_{{M_{4} }}^{2}$ are normal, then one gets that: (a) variables related to MM1 and MM2, i.e., $x_{1}$, $x_{2}$, $x_{3}$, $x_{4}$, and $x_{5}$, may be faulty; (b) all variables related to MM3 and MM4, i.e., $x_{1}$, $x_{4}$, $x_{5}$, $x_{6}$, and $x_{7}$, are normal; (c) $x_{3}$ must be faulty because it is the only common variable shared by MM1 and MM2, and *x*_2_ may also be faulty because we have no more information for judging it.
- For the $SPE_{M}$ index, when $SPE_{{M_{i} }}$ is faulty, then the correlation between all variables in MMi maybe faulty. For example, in the mathematical model in Fig. 3, one obtains $SPE_{{M_{1} }} { = }\left( {x_{1} { + }x_{2} - x_{3} } \right)^{2} \approx 0$; when the correlation between $x_{1}$, $x_{2}$, and *x*_3_ changes to $x_{1} - x_{2} = x_{3}$ or $x_{1} + 2 * x_{2} = x_{3}$, then $SPE_{{M_{1} }} = \left( {x_{1} + x_{2} - x_{3} } \right)^{2} \ne 0$ and $SPE_{{M_{i} }}$ alarms the fault.
- When a fault occurs in variables not belonging to the minimalist module, such as *x*_8_ and $x_{9}$, then they can only be handled with the detection result of the independent module, i.e., the contribution $Con T_{j}^{2}$.

## Simulation study of MMA

This section aims to study the performance of MMA through simulation tests, and compare it with PCA and mutual information–multiblock PCA (MI-MBPCA)^31^. MI-MBPCA employs mutual information to divide the block automatically and hence it does not need the process prior knowledge for block division. The test model is shown below:

$$\left\{ {\begin{array}{*{20}l} {x_{1} = N_{1} + 0.01 \times \omega_{1} } \hfill \\ {x_{2} = N_{2} + 0.01 \times \omega_{2} } \hfill \\ {x_{3} = x_{1} + x_{2} + 0.01 \times \omega_{3} } \hfill \\ {x_{4} = N_{3} + 0.01 \times \omega_{4} } \hfill \\ {x_{5} = x_{3} + x_{5} + 0.01 \times \omega_{5} } \hfill \\ {x_{6} = x_{5} + x_{1} + 0.01 \times \omega_{6} } \hfill \\ {x_{7} = x_{4} + 0.01 \times \omega_{7} } \hfill \\ {x_{8} = N_{4} + 0.01 \times \omega_{8} } \hfill \\ {x_{9} = N_{5} + 0.01 \times \omega_{9} } \hfill \\ \end{array} } \right..$$

Random variables *N*_*i*_ and $\omega_{i}$ follow the standard Gaussian distribution, and $\omega_{i}$ indicates the process noise. Approximately 10,000 normal observations are produced for offline modeling.

After data normalization, the training data are adjusted to zero-mean and unit-variance. Then the normalized data are processed by MMA. The matrix ${\tilde{\mathbf{P}}}_{r}$ is obtained as follows:

$${\tilde{\mathbf{P}}}_{r} { = }\left[ {\begin{array}{*{20}r} \hfill 0 & \hfill {0.53} & \hfill 0 & \hfill {0.39} \\ \hfill 0 & \hfill {0.50} & \hfill 0 & \hfill 0 \\ \hfill 0 & \hfill { - 0.68} & \hfill {0.60} & \hfill 0 \\ \hfill {0.69} & \hfill 0 & \hfill {0.39} & \hfill 0 \\ \hfill 0 & \hfill 0 & \hfill { - 0.70} & \hfill {0.45} \\ \hfill 0 & \hfill 0 & \hfill 0 & \hfill { - 0.80} \\ \hfill { - 0.72} & \hfill 0 & \hfill 0 & \hfill 0 \\ \hfill 0 & \hfill 0 & \hfill 0 & \hfill 0 \\ \hfill 0 & \hfill 0 & \hfill 0 & \hfill 0 \\ \end{array} } \right]$$.

Thus, MMA successfully obtains four minimalist modules: $\left\{ {x_{1} } \quad {x_{2} } \quad {x_{3} } \right\}$, $\left\{ {x_{3} } \quad {x_{4} } \quad {x_{5} } \right\}$, $\left\{ {x_{1} } \quad {x_{5} } \quad{x_{6} } \right\}$, and $\left\{ {x_{4} } \quad {x_{7} } \right\}$. Then, the independent module is $\left\{ {x_{8} } \quad {x_{9} } \right\}$.

And MI-MBPCA divides the process variables into the following 5 blocks: $\left\{ {x_{1} } \right\}$, $\left\{ {x_{2}} \quad {x_{3}} \quad {x_{5}} \quad{x_{6}} \right\}$, $\left\{ {x_{4} } \quad {x_{7} } \right\}$, $\left\{ {x_{8} } \right\}$, $\left\{ {x_{9} } \right\}$, which is not consistent with the process model because $x_{1}$ is correlated with both $x_{3}$ and $x_{6}$ but they do not belong to same block.

To compare the monitoring performance between MMA, PCA and MI-MBPCA, five test data sets are generated. Each data set contains 960 samples, and the fault occurs at the 160th sample point. The occurred faults are of the following five types:

Fault 1: a step change with amplitude of 5 in *x*_1_;

Fault 2: term *N*_2_ in the expression of *x*_2_ changes to $3 * N_{2}$;

Fault 3: a step change with amplitude of 0.2 in *x*_3_;

Fault 4: term $x_{3} + x_{4}$ in the expression of $x_{5}$ changes to $x_{3} + 2 * x_{4}$;

Fault 5: a step change with amplitude of 5 in $x_{8}$.

The detection results are listed in Table 1. The false alarm rate is calculated as $\frac{\text{the number of faults detected before 160}}{160}$ and the detection rate is calculated as $\frac{\text{the number of faults detected between 161 and 960}}{800}$. In this study, all control limits are based on a probability of 99% and the best result is marked in bold.

**Table 1:** False alarm rates (%) and detection rates (%) of the principal component analysis (PCA) method, the mutual information–multiblock PCA (MI-MBPCA), and the minimalist module analysis (MMA) method.

Method	PCA		MI-MBPCA	MMA		
Index	$T^{2}$	*SPE*	$DR$	$T_{I}^{2}$	$T_{M}^{2}$	*SPE*_*M*_
False alarm rate	1.9	3.1	0.6	1.9	1.9	0.0
Detection rate						
Fault 1	95.8	5.3	89.3	0.8	**99.0**	0.4
Fault 2	29.8	1.3	12.5	0.8	**38.4**	0.4
Fault 3	1.3	0.8	0.1	0.8	1.9	**93.8**
Fault 4	1.1	4.7	5.25	1.4	1.5	**90.3**
Fault 5	33.9	94.6	95.8	**97.8**	1.0	0.4

As shown in Table 1, the performance of MMA is better than that of PCA and MI-MBPCA for all five faults. Because MMA divides the whole process data into several minimalist modules and an independent module, and the noise in each variable will not disturb the unrelated modules, MMA is more robust to process noise than PCA. For MI-MBPCA, because each variable only belongs to one block and the rest blocks may lose key information, the models of blocks maybe biased. One interesting finding in Table 1 is that MMA can successfully detect faults 3 and 4 while PCA fails. The reason for this phenomenon is that PCA monitors the complex correlations between all variables together while MMA monitors each strong correlation (one minimalist module) independently; therefore, MMA is very sensitive to changes in specific correlations.

**Figure 4:**
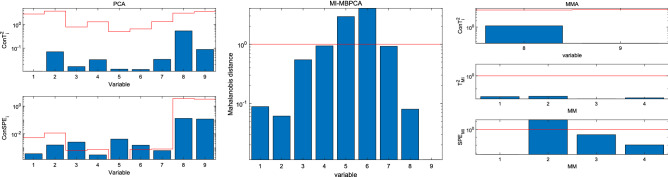
Fault localization for fault 3.

**Figure 5:**
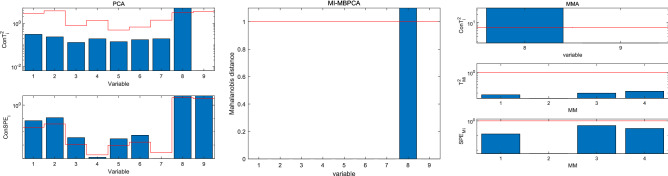
Fault localization for fault 5.

The fault localization results of the two algorithms for faults 3 and 5 are shown in Figs. 4 and 5, respectively. In Fig. 4, for PCA, $Con SPE_{3}$, $Con SPE_{5}$, and $Con SPE_{6}$ alarm the fault, and we cannot locate the fault source. For MI-MBPCA, because $x_{6}$ is influenced by $x_{5}$, both variables alarm the fault and we cannot locate the fault source. For MMA, all $ConT_{i}^{2}$ and $T_{{M_{i} }}^{2}$ indices are normal, which means that all variables in the independent module are normal and all variables in the minimalist modules fluctuate within the normal range; because $SPE_{{M_{2} }}$ signals a fault alarm, one finds that the correlations between $x_{1}$, $x_{2}$, and $x_{3}$ are changed.

In Fig. 5, although a fault occurs in $x_{8}$, most $Con SPE_{i}$ indices in PCA signal a fault alarm, and we cannot locate the fault source. For MI-MBPCA, it can successfully locate the fault source. However, because MI-MBPCA fails in detecting fault 5, and hence the fault localization step is skipped, as such, MI-MBPCA also fails in locating the fault source. For MMA, all $ConT_{i}^{2}$ and $SPE_{{M_{i} }}$ are normal, and hence one finds that the fault is not in the minimalist modules; only $Con T_{8}^{2}$ signals a fault alarm, and hence MMA successfully locates the faulty variable $x_{8}$.

## Fault detection in the Tennessee Eastman process

**Figure 6:**
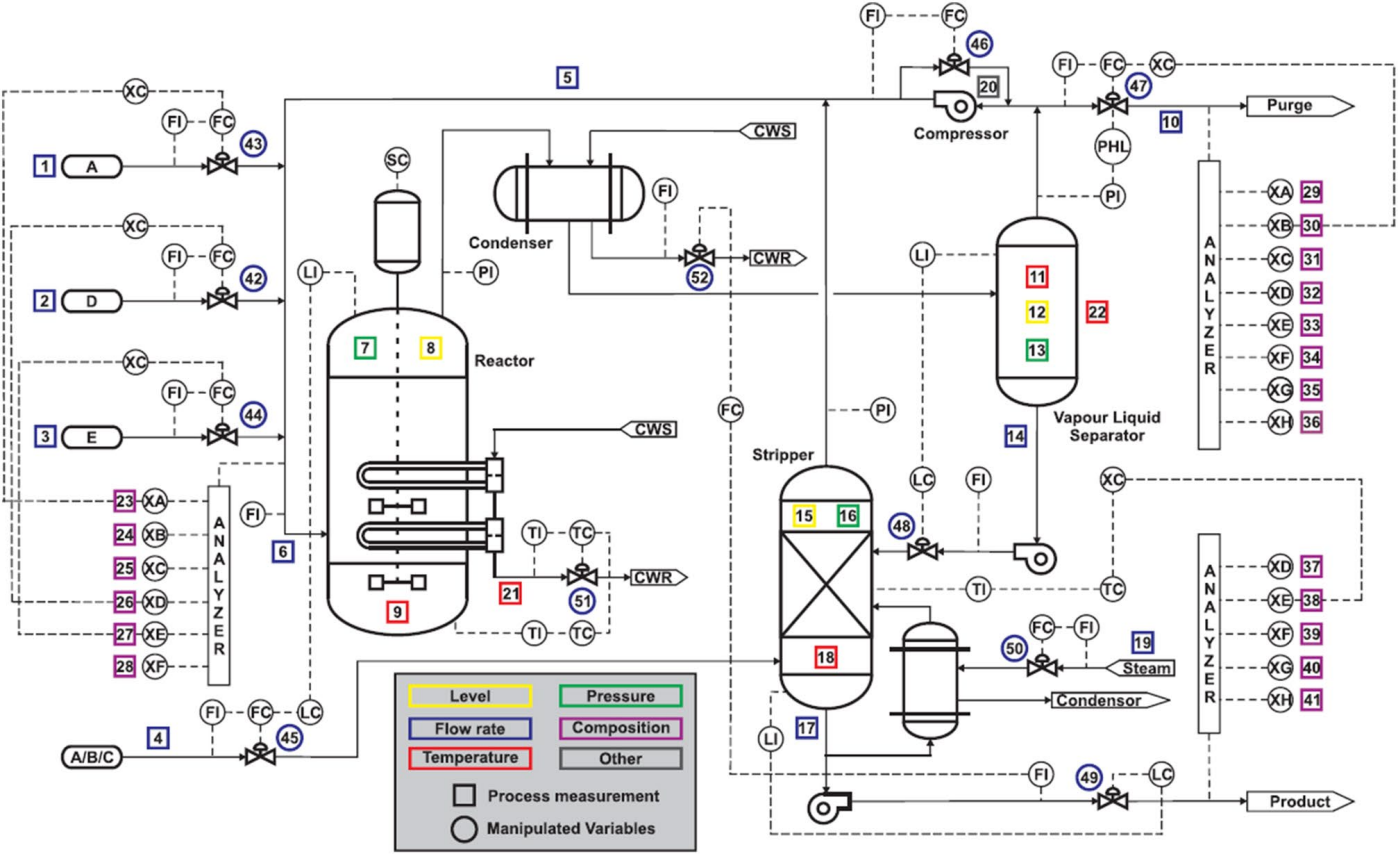
Schematic of the Tennessee Eastman process^33^.

The Tennessee Eastman (TE) process^32^ simulation is the most widely used simulation model to test the MSPM methods, which is outlined in Fig. 6. The TE process uses 12 manipulated variables, 22 continuous process measurements, and 19 composition measurements sampled less frequently to simulate a classical chemical process. Because the 19 composition measurements are difficult to measure in real time and one manipulated variable, i.e., the agitation speed, is not manipulated, this study only monitors the other 22 measurements and 11 manipulated variables, as listed in Table 2. Twenty-one programmed faults that are introduced in the TE process are listed in Table 3. In this study, 960 normal samples are adopted as training data to construct the monitoring models. Each testing data set contains 960 samples, and fault occurs at the 161st sample.

**Table 2:** Monitored variables in the Tennessee Eastman process^33^.

Variable	
1 A feed (stream 1)	18 Stripper temperature
2 D feed (stream 2)	19 Stripper steam flow
3 E feed (stream 3)	20 Compressor work
4 Total feed (stream 4)	21 Reactor cooling water outlet temperature
5 Recycle flow (stream 8)	22 Separator cooling water outlet temperature
6 Reactor feed rate (stream 6)	23 D feed flow valve (stream 2)
7 Reactor pressure	24 E feed flow valve (stream 3)
8 Reactor level	25 A feed flow valve (stream 1)
9 Reactor temperature	26 Total feed flow valve (stream 4)
10 Purge rate (stream 9)	27 Compressor recycle valve
11 Product separator temperature	28 Purge valve (stream 9)
12 Product separator level	29 Separator pot liquid flow valve (stream 10)
13 Product separator pressure	30 Stripper liquid product flow valve (stream 11)
14 Product separator under flow (stream 10)	31 Stripper steam valve
15 Stripper level	32 Reactor cooling water flow
16 Stripper pressure	33 Condenser cooling water flow
17 Stripper underflow (stream 11)

**Table 3:** Descriptions of faults in the Tennessee Eastman process^33^.

No.	Description	Type
1	Feed ratio of A/C, composition constant of B (stream 4)	Step
2	Composition of B, ratio constant of A/C (stream 4)	Step
3	Feed temperature of D (stream 2)	Step
4	Inlet temperature of reactor cooling water	Step
5	Inlet temperature of condenser cooling water	Step
6	Feed loss of A (stream 1)	Step
7	Header pressure loss of C—reduced availability (stream 4)	Step
8	Feed composite of A, B, and C (stream 4)	Random variation
9	Feed temperature of D (stream 2)	Random variation
10	Feed temperature of C (stream 4)	Random variation
11	Inlet temperature of reactor cooling water	Random variation
12	Inlet temperature of condenser cooling water	Random variation
13	Reaction kinetics	Slow drift
14	Valve of reactor cooling water	Sticking
15	Valve of condenser cooling water	Sticking
16–20	Unknown	Unknown
21	The valve for stream 4 was fixed at the steady-state position	Constant position

In this section, we compare MMA with PCA, MI-MBPCA, Deep principal component analysis (DePCA)^34^, and kernel dynamic PCA (KDPCA)^35^; the latter two methods are improved versions of PCA. The detection results of the four methods are listed in Table 4. The false alarm rate is calculated as the $\frac{\text{the number of faults detected before 160}}{160}$, and the detection rate is calculated as $\frac{\text{the number of faults detected between 161 and 960}}{800}$. In this study, all control limits are based on a probability of 99% and the best result is marked in bold.

**Table 4:** False alarm rates (%) and detection rates (%) of the four fault detection methods.

Method	PCA		DePCA		KDPCA		MI-MBPCA	MMA		
Index	$T^{2}$	*SPE*	_*ET*2_	_*ESPE*_	$T^{2}$	*SPE*	$DR$	$T_{I}^{2}$	$T_{M}^{2}$	*SPE*_*M*_
False alarm rate	0.5	1.4	6.1	11.5	11.21	4.05	1.25	0.8	1.3	0.2
**Detection rate**										
Fault 1	99.1	99.9	99.1	**100.0**	99.0	99.6	99.9	44.6	**100.0**	0.0
Fault 2	98.4	95.8	98.5	98.0	98.3	96.6	98.0	74.4	**98.6**	0.0
Fault 3	0.9	2.6	**17.6**	17.4	0.9	3.1	0.8	1.9	6.6	1.8
Fault 4	20.9	**100.0**	78.3	**100.0**	20.2	99.9	**100.0**	0.3	**100.0**	0.9
Fault 5	24.3	20.9	38.8	45.0	24.0	24.8	23.5	14.0	33.0	**100.0**
Fault 6	99.1	**100.0**	99.4	**100.0**	98.9	99.9	**100.0**	93.9	**100.0**	**100.0**
Fault 7	**100.0**	**100.0**	**100.0**	**100.0**	99.9	99.9	**100.0**	**100.0**	45.6	3.6
Fault 8	96.9	83.6	97.5	98.3	96.8	93.0	97.8	60.8	**98.4**	3.0
Fault 9	1.8	1.8	16.9	**14.0**	1.5	3.1	2.5	1.5	6.0	1.3
Fault 10	29.9	25.8	57.1	58.1	29.5	27.6	41.8	6.8	**88.5**	0.1
Fault 11	40.6	74.9	86.3	85.0	40.5	74.9	82.5	0.9	**89.4**	1.1
Fault 12	98.4	89.5	**99.6**	99.3	98.3	93.4	99.0	66.1	**99.6**	52.0
Fault 13	93.6	95.3	94.4	95.1	93.5	95.0	95.4	66.5	**95.6**	22.9
Fault 14	99.3	**100.0**	**100.0**	**100.0**	99.1	99.9	99.9	0.3	**100.0**	0.0
Fault 15	1.4	3.0	17.8	**19.6**	1.3	3.4	2.5	1.6	11.6	2.0
Fault 16	13.5	27.4	43.5	57.4	13.7	27.8	27.1	3.6	**91.9**	74.1
Fault 17	76.4	95.4	91.6	94.4	76.5	94.8	93.5	1.0	**97.1**	0.1
Fault 18	89.3	90.1	92.1	**92.0**	89.3	90.3	89.6	88.1	91.0	83.9
Fault 19	11.0	12.5	68.8	68.9	8.7	21.0	13.8	1.6	**90.4**	48.3
Fault 20	31.8	49.8	63.5	61.8	31.2	50.8	57.4	2.4	**83.9**	81.0
Fault 21	39.3	47.3	54.6	61.8	35.3	50.1	47.4	39.8	**66.3**	0.6

As shown in Table 4, we find that MMA, MI-MBPCA, and PCA achieve similar false alarm rates, and their values are much lower than those of the two improved PCA methods (over 10%). For fault detection rates, MMA achieves the best results in 17 of the 21 faults; as for the remaining 4 faults, MMA’s detection rates are not as high as those of DePCA only because DePCA sacrifices the false alarm rate. An eye-catching result is obtained in the case of fault 5: the detection rates of the compared methods are generally below 50%, whereas MMA achieves a 100.0% detection rate, which indicates the superiority of MMA. In addition, the performance of MMA in faults 10, 16, 19, and 20 is much better than that of the other four methods.

As the papers that proposed DePCA and KDPCA did not give a description of the contribution plot construction, we only compare the fault localization ability between PCA, MI-MBPCA, and MMA. The matrix ${\tilde{\mathbf{P}}}_{r}$ of MMA is shown in Table 5.

**Table 5:** Matrix ${\tilde{\mathbf{P}}}_{r}$ for the Tennessee Eastman process.

Variable	MM													
	1	2	3	4	5	6	7	8	9	10	11	12	13	14
1	**− 0.1**	0.0	**0.3**	**− 0.3**	**0.1**	0.0	0.0	**0.3**	0.0	**− 0.2**	**− 0.2**	**0.3**	0.0	0.0
2	**0.1**	0.0	**0.1**	**0.1**	**0.3**	0.0	0.0	0.0	**− 0.2**	0.0	0.0	**0.2**	**− 0.1**	**0.1**
3	0.0	0.0	0.0	0.0	0.0	0.0	0.0	0.0	0.0	0.0	0.0	0.0	0.0	0.0
4	0.0	0.0	0.0	0.0	0.0	0.0	0.0	0.0	0.0	0.0	0.0	0.0	0.0	0.0
5	0.0	0.0	**0.1**	0.0	0.0	0.0	0.0	0.0	0.0	0.0	0.0	0.0	0.0	**0.1**
6	0.0	0.0	0.0	0.0	0.0	0.0	0.0	0.0	0.0	0.0	0.0	0.0	0.0	0.0
7	**− 0.3**	**− 0.6**	**0.2**	**− 0.3**	0.0	**0.4**	0.0	**0.1**	**− 0.1**	0.0	0.0	0.0	0.0	**0.4**
8	0.0	0.0	0.0	0.0	0.0	0.0	0.0	0.0	0.0	0.0	0.0	0.0	0.0	0.0
9	**0.5**	0.0	0.0	0.0	0.0	**0.3**	0.0	**− 0.3**	0.0	0.0	0.0	0.0	**0.4**	0.0
10	**− 0.1**	0.0	**− 0.2**	0.0	**0.1**	0.0	0.0	**0.2**	**0.1**	**− 0.2**	0.0	0.0	**− 0.2**	**0.1**
11	0.0	**0.1**	**0.2**	0.0	**0.1**	0.0	**0.4**	**0.2**	0.0	**− 0.4**	**0.1**	**0.3**	0.0	0.0
12	0.0	0.0	0.0	**0.1**	0.0	0.0	0.0	**0.2**	0.0	0.0	0.0	0.0	0.0	0.0
13	0.0	**0.5**	**0.3**	0.0	**0.3**	0.0	0.0	**− 0.3**	**− 0.2**	0.0	**− 0.1**	**− 0.5**	**0.4**	**− 0.1**
14	0.0	0.0	0.0	0.0	0.0	0.0	0.0	0.0	0.0	0.0	0.0	0.0	0.0	0.0
15	**− 0.1**	0.0	0.0	0.0	0.0	0.0	0.0	0.0	0.0	0.0	**0.1**	0.0	0.0	**0.4**
16	**0.5**	**0.5**	**− 0.1**	**− 0.1**	**− 0.4**	**0.1**	**− 0.3**	**0.4**	**0.5**	**0.4**	0.0	**0.2**	**− 0.2**	**0.3**
17	0.0	0.0	0.0	0.0	0.0	0.0	**− 0.4**	0.0	**− 0.5**	0.0	0.0	0.0	0.0	0.0
18	0.0	**0.2**	0.0	**− 0.2**	**0.2**	**0.6**	**− 0.4**	**− 0.1**	0.0	**− 0.1**	**0.1**	0.0	**0.1**	0.0
19	**0.3**	0.0	**0.6**	0.0	**− 0.4**	**− 0.1**	**− 0.1**	0.0	0.0	**0.4**	**0.5**	0.0	**− 0.2**	**0.5**
20	0.0	0.0	**− 0.1**	**0.6**	**0.1**	**− 0.5**	**0.3**	0.0	0.0	**− 0.6**	0.0	**0.1**	**0.4**	**− 0.4**
21	**0.3**	**0.1**	**0.2**	**0.2**	**0.5**	0.0	0.0	0.0	**− 0.3**	0.0	**− 0.2**	**0.5**	0.0	0.0
22	**0.1**	0.0	0.0	**0.1**	**0.1**	0.0	**− 0.4**	**− 0.1**	0.0	**0.3**	**− 0.1**	0.0	**− 0.1**	0.0
23	0.0	0.0	0.0	**0.2**	**0.3**	**− 0.1**	0.0	0.0	**− 0.1**	0.0	**− 0.1**	**0.2**	**− 0.1**	**− 0.1**
24	0.0	0.0	0.0	**0.1**	0.0	0.0	0.0	0.0	0.0	**− 0.1**	**0.1**	0.0	0.0	0.0
25	0.0	**− 0.2**	**− 0.4**	**0.4**	0.0	**− 0.1**	0.0	**− 0.4**	**− 0.1**	0.0	**0.4**	**− 0.2**	**− 0.1**	0.0
26	0.0	0.0	0.0	0.0	0.0	0.0	0.0	0.0	0.0	0.0	0.0	0.0	0.0	0.0
27	0.0	**− 0.2**	**− 0.2**	**− 0.2**	0.0	**0.2**	**0.2**	0.0	0.0	0.0	**0.2**	**0.3**	**− 0.5**	0.0
28	0.0	0.0	**0.1**	0.0	**− 0.2**	**− 0.1**	0.0	**− 0.1**	0.0	**0.1**	0.0	0.0	**0.2**	**− 0.1**
29	0.0	0.0	0.0	**− 0.1**	0.0	0.0	0.0	**− 0.2**	0.0	0.0	0.0	0.0	0.0	0.0
30	**0.1**	0.0	0.0	0.0	0.0	0.0	0.0	0.0	0.0	0.0	**− 0.1**	0.0	0.0	**− 0.4**
31	0.0	0.0	**− 0.2**	**− 0.2**	**0.1**	**− 0.1**	0.0	**0.2**	0.0	**0.2**	**− 0.6**	0.0	0.0	0.0
32	**− 0.5**	0.0	0.0	0.0	0.0	**− 0.3**	0.0	**0.3**	0.0	0.0	0.0	0.0	**− 0.4**	0.0
33	0.0	0.0	0.0	0.0	0.0	0.0	**− 0.4**	0.0	**− 0.5**	0.0	0.0	0.0	0.0	0.0

Significant values are in [bold].

**Figure 7:**
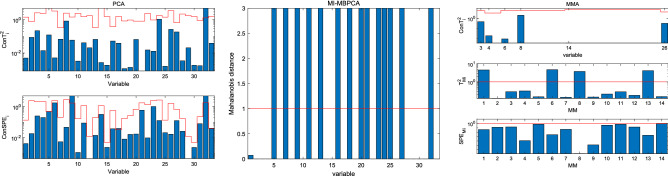
Fault localization for fault 4.

Figure 7 shows the fault localization results of fault 4. According to Table 3, fault 4 is a step change in inlet temperature of reactor cooling water. As depicted in Fig. 6, the reactor temperature (variable 9 in Table 2) changes, and hence the reactor cooling water flow (variable 32 in Table 2) also changes to compensate for the temperature change. For PCA, $Con SPE_{9}$, $Con SPE_{32}$, and $Con T_{32}^{2}$ signal a fault alarm; for MI-MBPCA, about 14 variables alarm the fault and it fails in locating the fault source; for MMA, $T_{{M_{1} }}^{2}$, $T_{{M_{6} }}^{2}$, $T_{{M_{8} }}^{2}$, and $T_{{M_{13} }}^{2}$ signal a fault alarm based on the fault localization rules presented in “Monitoring indices construction” section, and then one finds that variables 9 and 32 are faulty. Both PCA and MMA can locate this fault. Different from the contribution plot method of PCA, all $SPE_{{M_{i} }}$ of MMA are normal, which tells the engineers that the correlation between variables have not changed, and hence the fault source is the change in amplitude of some variables. Thus, it can be seen that, compared with PCA, MMA can provide more useful information for fault localization.

## Conclusions

In this study, a new MSPM called MMA was proposed to overcome the shortcoming of the traditional MSPM method in handling the redundant correlations among process variables.

The superiority of MMA was verified by several simulation tests. It achieved much better detection performance for five different types of faults on a mathematical model test, and two of which could not be detected by PCA and MI-MBPCA. MMA also had a better performance than other improved MSPM algorithms for 17 of the 21 faults in the Tennessee Eastman process.

MMA is a completely new method, and hence much work can be done based on it. First, we can combine it with the traditional nonlinear, dynamic, robust strategy to improve its fault detection ability. We can also combine it with the traditional contribution plot method to improve its fault localization ability. Moreover, we can combine it with the key performance indicator^14^ monitoring strategy. All of these investigations will be part of our future work.
